# Meningococcal Disease in US Military Personnel before and after Adoption of Conjugate Vaccine

**DOI:** 10.3201/eid2211.150498

**Published:** 2016-11

**Authors:** Michael D. Decker

**Affiliations:** Sanofi Pasteur, Swiftwater, Pennsylvania, USA; Vanderbilt University School of Medicine, Nashville, Tennessee, USA

**Keywords:** meningococcal infections, vaccination, epidemiology, epidemiologic methods, bacteria, US military, vaccines, meningitis

**To the Editor:** In their recent letter (*1*), Broderick et al. provided useful information about the remarkable declines in incidence of meningococcal disease among active-duty US military personnel since the early 1970s, when meningococcal vaccination began within that population. The authors reported that the incidence of meningococcal disease from vaccine-covered serogroups was 0.183 cases/100,000 persons during 2006–2013 among persons vaccinated with quadrivalent conjugate meningococcal vaccine (MCV-4), compared with 0.307 cases/100,000 persons during 2000–2013 among persons vaccinated with quadrivalent polysaccharide meningococcal vaccine (MPSV-4). They stated that, because these rates did not differ significantly, case rates were similar in personnel vaccinated with MCV-4 and MPSV-4. Although statistically correct, this comment might mislead the unwary reader.

The absence of a significant difference does not necessarily mean that the 2 vaccines have similar effectiveness. The incidence rate of meningococcal disease was 68% higher ([0.307–0.183] × 100/0.183) during the period of MPSV-4 use than during the period of MCV-4 use. If the same findings arose in a study of sufficient size to achieve statistical significance, this difference would be considered of substantial clinical importance. A happy consequence of the long-term temporal trends in meningococcal incidence and the success of these vaccines is that the incidence of meningococcal disease is now sufficiently reduced that even the very large active-duty population is too small to provide the statistical power to declare these 2 different incidence rates as being statistically different.

The trends reported by Broderick et al. have continued. During 2006–2014, the incidence of meningococcal disease caused by vaccine-covered serogroups among US military recipients of MCV-4 fell to 0.146 per 100,000 person-years, whereas MPVS-4–related incidence did not change (M.P. Broderick, pers. comm.). Furthermore, through July 2016, the US military has not seen a case from a covered serogroup since 2011 among recipients of MCV-4. Even with these additional data, however, the difference between MCV-4 and MPSV-4 does not achieve statistical significance (M.P. Broderick, pers. comm.).
